# Methylene Blue-Mediated Photodynamic Therapy Induces Macrophage Apoptosis via ROS and Reduces Bone Resorption in Periodontitis

**DOI:** 10.1155/2019/1529520

**Published:** 2019-08-14

**Authors:** Chunlan Jiang, Wenyi Yang, Chengyi Wang, Wei Qin, Jiajun Ming, Manman Zhang, Haixin Qian, Ting Jiao

**Affiliations:** ^1^Department of Prosthodontics, Shanghai Ninth People's Hospital, College of Stomatology, Shanghai Jiao Tong University School of Medicine, Shanghai 200011, China; ^2^Shanghai Key Laboratory of Stomatology & Shanghai Research Institute of Stomatology, National Clinical Research Center for Oral Diseases, Shanghai 200011, China; ^3^Department of Oral Maxillofacial-Head and Neck Oncology, Shanghai Ninth People's Hospital, College of Stomatology, Shanghai Jiao Tong University School of Medicine, Shanghai 200011, China

## Abstract

**Aim:**

To investigate whether methylene blue-mediated photodynamic therapy (MB-PDT) can affect the “fate” of macrophages *in vitro* or in periodontitis tissues and to explore the potential mechanism.

**Methods:**

For *in vitro* treatments, THP-1 macrophages were divided into three experimental groups: C/control, no treatment; MB, methylene blue treatment; and MB-PDT, MB and laser irradiation treatment. Then, apoptosis and apoptosis-related proteins were detected in each group. For *in vivo* treatments, periodontitis was ligature-induced in the first molars of the bilateral maxilla in 12 Sprague Dawley (SD) rats. After six weeks, the ligatures were removed and all the induced molars underwent scaling and root planning (SRP). Then, the rats were divided into three groups according to the following treatments: SRP, saline solution; MB, phenothiazinium dye; and MB-PDT, MB and laser irradiation. Apoptotic macrophages, inflammation levels, and alveolar bone resorption in the periodontal tissues of rats were analyzed in each group.

**Results:**

*In vitro*, flow cytometry analysis demonstrated that 10 *μ*M MB and 40 J/cm^2^ laser irradiation maximized the apoptosis rate (34.74%) in macrophages. Fluorescence probe and Western blot analyses showed that MB-PDT induced macrophage apoptosis via reactive oxygen species (ROS) and the mitochondrial-dependent apoptotic pathway. Conversely, the addition of exogenous antioxidant glutathione (GSH) and the pan-caspase inhibitor Z-VAD-FMK markedly reduced the apoptotic response in macrophages. *In vivo*, immunohistochemistry, histology, radiographic, and molecular biology experiments revealed fewer infiltrated macrophages, less bone loss, and lower IL-1*β* and TNF-*α* levels in the MB-PDT group than in the SRP and MB groups (*P* < 0.05). Immunohistochemistry analysis also detected apoptotic macrophages in the MB-PDT group.

**Conclusion:**

MB-PDT could induce macrophage apoptosis *in vitro* and in rats with periodontitis. This may be another way for MB-PDT to relieve periodontitis in addition to its antimicrobial effect. Meanwhile, MB-PDT induced apoptosis in THP-1 macrophages via the mitochondrial caspase pathway.

## 1. Introduction

Periodontal disease is the result of the collapse of tooth-supporting structures by the local action of periodontopathogenic microorganisms, which release inflammatory substances that severely injure periodontal tissues. Tissue destruction can also be caused by inflammatory and immunologic responses of the host [1]. As critical inflammatory cells, macrophages play an important role during the occurrence and development of periodontitis. As innate immune cells, macrophages exert a defensive function and maintain the homeostasis of periodontal tissue [2]. However, macrophages can also interact with local tissue cells, including gingival epithelial cells, fibroblasts, endothelial cells, and osteoblasts, and secrete IL-1, IL-6, TNF-*α*, PGE2, and other cytokines, resulting in inflammatory lesions in periodontal tissue and absorption of alveolar bone [3, 4]. Gemmell found that the number of macrophages in gingival tissues of patients with chronic periodontitis was considerably increased compared with that in normal gingival tissues [5]. Similarly, Jagannathan [6] detected extensive macrophage infiltration in the gingival crevicular fluid of patients and animals with periodontitis and investigated several proinflammatory factors and bone resorption factors, such as IL-1*β* and TNF-*α*. Therefore, in addition to eliminating bacteria, reducing the number of overinvasive macrophages is also critical for periodontitis treatment.

The standard management of periodontal diseases focuses on infection control, detoxification of dental surfaces, regeneration of lost tissues, and plaque-control regimens via mechanical debridement (such as scaling and root planning (SRP)) [7]. Other therapeutic modalities, such as antiseptics and/or antibiotics, laser therapy, guided bone regeneration, and air-polishing, have also been proposed [8–11]. However, the outcomes of clinical treatment remain less than ideal. Recently, photodynamic therapy (PDT) has been presented as a promising treatment strategy for periodontitis [12–15].

The effects of PDT are based on three components: light, a photoactive agent (photosensitizer), and the presence of oxygen [16]. Photodynamic therapy under high-energy single-frequency light (e.g., diode lasers) in combination with photosensitizers (PSs) generates reactive oxygen species, such as free radicals and/or singlet oxygen, which are toxic to microorganisms [17]. The most widely used PSs in dental clinical practice are phenothiazine dyes, such as methylene blue (MB) and toluidine blue (TBO) [18]. Recently, almost all existing studies about PDT have focused on pathogenic bacteria to reduce the level of inflammation in periodontal tissues [19–21]. However, no study has explored the role of inflammatory cells under PDT in periodontitis.

The purpose of this study is to investigate whether MB-mediated PDT can affect the “fate” of macrophages in periodontitis tissues. The hypothesis is that MB-mediated PDT can deplete hyperproliferating macrophages and inhibit the progression of periodontitis. The underlying mechanism on macrophages will be explored simultaneously.

## 2. Materials and Methods

### 2.1. Reagents

The photosensitizer MB used for PDT was acquired from Sangon Biotech (Shanghai, China). The 660 nm light (300 mW) was produced by a red diode laser (Lingyue Photoelectric Technology, Hangzhou, China). A laser diode (LD) emitter was used to irradiate samples from a constant distance of approximately 7 cm, and the lens was adjusted to match the area of the light spot formed by irradiation with the area of the bottom of cell culture plates, including 96-well plates and 6-well plates.

### 2.2. Cell Culture

Human THP-1 monocytes (ATCC, Manassas, VA, USA) were cultured in RPMI-1640 medium (Sigma-Aldrich, USA) supplemented with 10% fetal bovine serum (BI1 Israel), 100 units/ml penicillin, and 100 units/ml streptomycin. Cultures were maintained at 37°C in a humidified incubator containing 5% CO_2_, and the medium was refreshed every 2-3 days. For the experiment, the cells were differentiated into macrophages by adding 100 ng/ml phorbol 12-myristate 13-acetate (Sigma-Aldrich, USA) for 48 h [22].

### 2.3. Cell Viability Assay

Cell viability was measured using the 3-(4,5-dimethylthiazol-2-yl)-2,5-diphenyl tetrazolium bromide (MTT) assay. Cells treated with phorbol 12-myristate 13-acetate (100 ng/ml) were seeded at a density of 5 × 10^5^ cells/ml into 96-well plates and incubated for 48 h at 37°C in the dark. The cells were then subjected to the following experimental treatments: (1) the cells were incubated with 5 *μ*M MB for 10 minutes and exposed to a 660 nm laser at 0-50 J/cm^2^ after changing the new complete culture medium and (2) the cells were incubated with different methylene blue concentrations (0-20 *μ*M) according to a previous study [23] for 10 minutes and exposed to a 660 nm laser at 40 J/cm^2^ after changing the medium. After 24 h of incubation following PDT, the medium was removed and 100 *μ*l of medium containing 0.5 mg/ml MTT without FBS was added to the cells, which were then incubated for 4 h at 37°C in the dark. Absorption at 490 nm was subsequently measured on a microplate reader (BioTek ELx800, BioTek, Winooski, VT, USA).

### 2.4. Cell Apoptosis Rate

The cell apoptosis rate was detected by Annexin V-FITC/propidium iodide (PI) flow cytometry. Cells were seeded at a density of 5 × 10^5^ cells/ml into 6-well plates, and then photosensitization experiments (MB ranging from 0 to 15 *μ*M with or without a laser providing 40 J/cm^2^ irradiation) were performed after 48 h when the cells had grown to 80-90% confluence. After MB-PDT treatment, macrophages were incubated for 6 h at 37°C. Apoptosis was then analyzed using a flow cytometer with Annexin V-FITC (fluorescein isothiocyanate) and PI staining. An apoptosis detection kit (YEASEN, Shanghai, China) was used in the present study. Briefly, cells from each sample were suspended in a mixture of 100 *μ*l of Annexin V-FITC binding buffer, 5 *μ*l of Annexin V-FITC, and 10 *μ*l of PI and were then incubated at room temperature (RT) for 15 minutes. Finally, an additional 400 *μ*l of Annexin V-FITC binding buffer was added, and the sample was placed on ice. The samples were analyzed using the Beckman Coulter CytoFlex S flow cytometer (SE, Becton Dickinson). The cell population was separated into three groups: live cells with a low level of fluorescence, early apoptotic cells with green fluorescence, and necrotic and advanced-stage apoptotic cells with both red and green fluorescence. From the Annexin V chart the following were observed: Living cells (Annexin V^−^/PI^−^) were observed in the left lower (LL) quadrant. Early apoptotic cells (Annexin V^+^/PI^−^) were observed in the right lower (LR) quadrant. Advanced apoptotic or necrotic cells (Annexin V^+^/PI^+^) were observed in the right upper (UR) quadrant. The left upper (UL) quadrant may contain nuclei, cells in late necrosis, or cellular debris (Annexin V^−^/PI^+^). According to some studies [24, 25], UR results plus LR results indicate overall apoptosis.

### 2.5. Measurement of Intracellular and Mitochondrial Reactive Oxygen Species (ROS) Generation

Cells were seeded in 6-well plates, and then photosensitization experiments (MB 10 *μ*M, laser 40 J/cm^2^) were performed after 48 h when the cells had grown to 80-90% confluence. Six hours after MB-PDT treatment, the cells were stained with 20 *μ*M DCFH-DA (YEASEN, Shanghai, China) to measure intracellular ROS, such as hydroxyl radicals and peroxynitrite anion, and 1 *μ*M MitoSOX (YEASEN, Shanghai, China) to measure mitochondrial ROS (superoxides) for 30 minutes at 37°C in a humidified atmosphere in the dark [26, 27]. The cells were then gently washed twice with phosphate-buffered saline (PBS). Both intracellular and mitochondrial ROS were identified using a fluorescence spectrophotometer (Zeiss, Gottingen, Germany). Both DCF (green fluorescence, 488 nm excitation wavelength) and MitoSOX (red fluorescence, 555 nm excitation wavelength) florescence intensities were analyzed using Zeiss CLSM software (ZEN 2009 Light Edition). The experiments were repeated three times independently.

### 2.6. Detection of Mitochondrial Membrane Potential (MMP)

MMP was assessed via a JC-1 fluorescent probe (Beyotime Biotechnology, China). Cells were seeded in 6-well plates, and then photosensitization experiments (MB 10 *μ*M, laser 40 J/cm^2^) were performed after 48 h when the cells had grown to 80-90% confluence. Six hours after MB-PDT treatment, the macrophages were incubated with JC-1 for 20 minutes at 37°C in the dark and were analyzed under a fluorescence microscope. Red-orange fluorescence is attributable to potential-dependent aggregation in mitochondria. Green fluorescence, which reflects the monomeric form of JC-1, appeared in the cytosol after mitochondrial membrane depolarization. Fluorescence intensity was measured with a fluorescence spectrophotometer (BioTek Cytation™ 3 Cell Imaging Multi-Mode Reader, BioTek, Winooski, VT, USA) at an excitation wavelength of 488 nm and emission wavelengths of 530 nm (green) and 590 nm (red) [28]. The experiments were repeated three times independently.

### 2.7. Detection of Mitochondrial Permeability Transition Pore (MPTP) Changes

Intracellular mitochondria were stained specifically with calcein AM through Co^2+^ quenching of cytosolic calcein fluorescence. Cells were seeded in 6-well plates, and photosensitization experiments (MB 10 *μ*M, laser 40 J/cm^2^) were performed after 48 h when the cells had grown to 80-90% confluence. Approximately 6 h after PDT, the cells were stained with 5 *μ*M calcein AM (Aladdin, Shanghai, China) in the presence of 5 mM cobalt chloride (Aladdin, Shanghai, China) in the dark for 20 minutes at 37°C. The cells were then carefully washed twice with PBS. Fluorescence was examined using a fluorescence microscope (Zeiss, Gottingen, Germany), and changes in the MPTP following PDT were also determined using the same method. Calcein AM fluorescence was measured at 488 nm excitation and 525 nm emission wavelengths. The experiments were repeated three times independently.

### 2.8. Western Blot Analysis

The following antibodies were used for Western blot analysis, including anti-cleaved caspase-9, anti-cytochrome c, anti-cleaved caspase-3, anti-poly (ADP ribose) polymerase (PARP), and anti-GAPDH. Cells were seeded in 6-well plates, and then photosensitization experiments (MB 10 *μ*M, laser 40 J/cm^2^) were performed after 48 h when the cells had grown to 80-90% confluence. Approximately 0, 3, 6, and 12 h after PDT, the cells were harvested. Subcellular fractionation (cytosolic versus mitochondrial) of samples was performed according to the instruction manual of the mitochondrial fractionation kit (Beyotime Biotechnology, China). In brief, cells were harvested, washed twice with ice-cold PBS, resuspended in ice-cold cytosolic buffer, and then incubated on ice for 15 minutes. The cells were homogenized on ice using 30 strokes with the homogenizer, and the nuclei and intact cells were removed by centrifugation at 3,000 rpm for 15 minutes at 4°C. The supernatant was collected and centrifuged at 12,000 rpm for 30 minutes at 4°C to obtain a cytosolic supernatant. The supernatant was centrifuged at 16,000 × *g* for 20 minutes at 4°C to remove any residual mitochondria. The bicinchoninic acid protein assay reagent (Beyotime Biotechnology, China) was used to measure the protein concentration in the lysate. Protein samples were mixed with a 5x sample loading buffer (10% SDS, 5% *β*-mercaptoethanol, 15% glycerol, 0.01% bromophenol blue, and 200 mM Tris-HCl, pH 6.7), separated on a 10-15% linear SDS-PAGE gel, and then transferred to polyvinylidene fluoride membranes (Thermo Fisher Scientific, USA). Then, the polyvinylidene fluoride membranes were blocked with 5% nonfat milk in Tris-buffered saline-Tween 20 (0.05%) at room temperature for 2 h. The membranes were subsequently probed with primary antibodies (against cytochrome c, cleaved caspase-9, cleaved caspase-3, PARP, and cleaved PARP; Cell Signaling Technology Inc., Danvers, MA, USA) overnight at 4°C. All primary antibodies were diluted 1 : 1,000. After three washes with Tris-buffered saline-Tween 20, the membranes were incubated with alkaline phosphatase-labeled secondary antibodies at room temperature for 1 h. The resultant immune complexes were detected with enhanced chemiluminescence reagents. All secondary antibodies were diluted 1 : 12,000. Anti-GAPDH (Cell Signaling Technology Inc.) was used to confirm equal cytosol loading [28].

### 2.9. Animals and Protocol for Experimental Periodontal Disease

This study was conducted using 12 three-month-old male Sprague Dawley (SD) rats. The animals were housed in plastic cages with access to food and water ad libitum. Before the surgical procedures, all animals were allowed to acclimatize to the laboratory environment for a period of 5 days. All protocols described below were approved by the Ninth People's Hospital Affiliated to Shanghai Jiao Tong University. Bilateral maxillary first molars of each animal were selected to undergo orthodontic ligature in the submarginal position to induce experimental periodontitis [29]. A mix of 10^9^ colony-forming units (CFU) of each periodontal pathogen, such as *Porphyromonas gingivalis* (Pg, ATCC 33277) and *Fusobacterium nucleatum* (Fn, ATCC 25586), was applied on the surface of the maxillary first molar teeth according to a previous study [30]. After six weeks of experimental periodontal disease induction, the ligature of the mandibular first molar was removed in all animals. The molars were subjected to SRP, which was performed by the same experienced operator. The 12 animals were randomly allocated to three groups (*n* = 4 per group): SRP, molars were subjected to SRP and irrigation with 0.1 ml of saline solution; MB, the molars were subjected to SRP and irrigation with 0.1 ml of MB (10 mg/ml) solution; and PDT, the molars were subjected to SRP and irrigation with 0.1 ml of MB (10 mg/ml) solution, followed by application of a low-intensity laser after 3 minutes. The laser was applied (approximately 100 J/cm^2^ at each site) circumferentially at six sites per molar for 1 minute. All animals were sacrificed 21 days after periodontal disease treatment once a week by administration of a lethal dose of glutaraldehyde (40 mg/kg).

### 2.10. Quantitative Real-Time Polymerase Chain Reaction (PCR)

Total RNA from rat periodontal tissue was prepared using a TRIzol reagent (Sigma-Aldrich, USA) according to the manufacturer's instructions. The quality of the total RNA was evaluated by measuring the A260/A280 nm ratio using a fluorospectrometer (NanoDrop ND-1000; Thermo Scientific, Wilmington, DE, USA). cDNA was synthesized using a reverse transcription kit (Takara Bio Inc., Japan) according to the manufacturer's instructions. Real-time PCR was performed using a StepOnePlus™ Real-Time PCR System (Thermo Scientific, USA). To control variability in amplification due to differences in starting mRNA concentrations, GAPDH was used as an internal control. The PCR primers are presented in Table 1.

### 2.11. Enzyme-Linked Immunosorbent Assay (ELISA)

After cutting rat periodontal tissues, the weight was measured and PBS was added. Then, samples were rapidly frozen with liquid nitrogen and maintained at 2-8°C. After the samples were thawed, PBS was added. The samples were homogenized by hand and centrifuged for 20 minutes at 2,000-3,000 rpm, and the supernatant was collected. TNF-*α* and IL-1*β* concentrations in rat periodontal tissue were estimated using a TNF-*α*/IL-1*β* ELISA kit (Youpeinuo Biotechnology, Shanghai, China) according to the manufacturer's instruction. In brief, the solution was diluted, and the sample was added to the standard (TNF-*α* density: 240 ng/l, 160 ng/l, 80 ng/l, 40 ng/l, and 20 ng/l; IL-1*β* density: 60 ng/l, 40 ng/l, 20 ng/l, 10 ng/l, and 5 ng/l). Then, 40 *μ*l of the diluted sample and 10 *μ*l of the test sample were added to the testing well. Subsequently, 50 *μ*l of HRP-conjugate reagent was added to each well. The sample was incubated for 30 minutes at 37°C, and each well was washed with Wash Buffer. Then, 50 *μ*l of Chromogen Solutions A and B was added into each well, and the solution was incubated for 10 minutes at 37°C. After Stop Solution was added, the optical density of each well was determined using a microplate reader set to 450 nm.

### 2.12. Microcomputed Tomography (CT) Analysis

The maxillary jaws were hemisected, and both halves of the block samples (*n* = 8) were fixed in 4% paraformaldehyde and subjected to routine microcomputed tomography (micro-CT) (LaTheta LCT-200, Aloka, Japan) to evaluate alveolar bone loss and for histological analysis. Three-dimensional images were acquired using the VGStudio MAX 2.2 (Volume Graphics, Germany). The distance between the cementoenamel junction and the alveolar bone crest was determined for the mesial root surface of the first molars [14]. Bone loss was measured in millimeters on each radiograph in mask mode three times by the same examiner.

### 2.13. Histology and Immunohistochemical Analyses

After micro-CT scanning, the maxillary jaws were decalcified in 15% EDTA solution, which was exchanged every other day for 1 month. Next, the samples were dehydrated, embedded in paraffin blocks, and cut into 4 *μ*m sections. After dewaxing and rehydration, the sections were stained with hematoxylin and eosin or subjected to immunohistochemical analysis with mouse polyclonal anti-CD68 (1 : 100, Cat number: ab955, Abcam, Cambridge, UK). A trained examiner who was blinded to the treatment examined the sections for immunohistochemical analyses. Each section was measured three times by the same examiner.

### 2.14. Terminal 2′-Deoxyuridine-5′-Triphosphate (dUTP) Nick End Labeling (TUNEL)

After dewaxing and rehydration, the sections were stained with mouse polyclonal anti-CD68 (1 : 100, Cat number: ab955, Abcam, Cambridge, UK) and terminal deoxynucleotidyl transferase dUTP nick end labeling (TUNEL) (YEASEN, Shanghai, China) to detect the apoptosis of macrophages in the periodontal tissues of the rats. In brief, the sections were first incubated with anti-CD68 and Cy3-AffiniPure goat anti-mouse IgG (H+L) and subsequently incubated with fluorescence-labeled dUTP. Nuclei was stained with DAPI. The apoptotic macrophages (green and red) were observed and analyzed using a fluorescence microscope.

### 2.15. Statistical Analysis

The results were presented as the mean ± standard deviation and statistically analyzed with one-way analysis of variance (ANOVA), followed by the least-significant difference (LSD) test or a *t*-test for paired samples. *P* values less than 0.05 were considered statistically significant.

## 3. Results

### 3.1. MB-PDT Induced Apoptosis in THP-1 Macrophages

**Figure 1:**
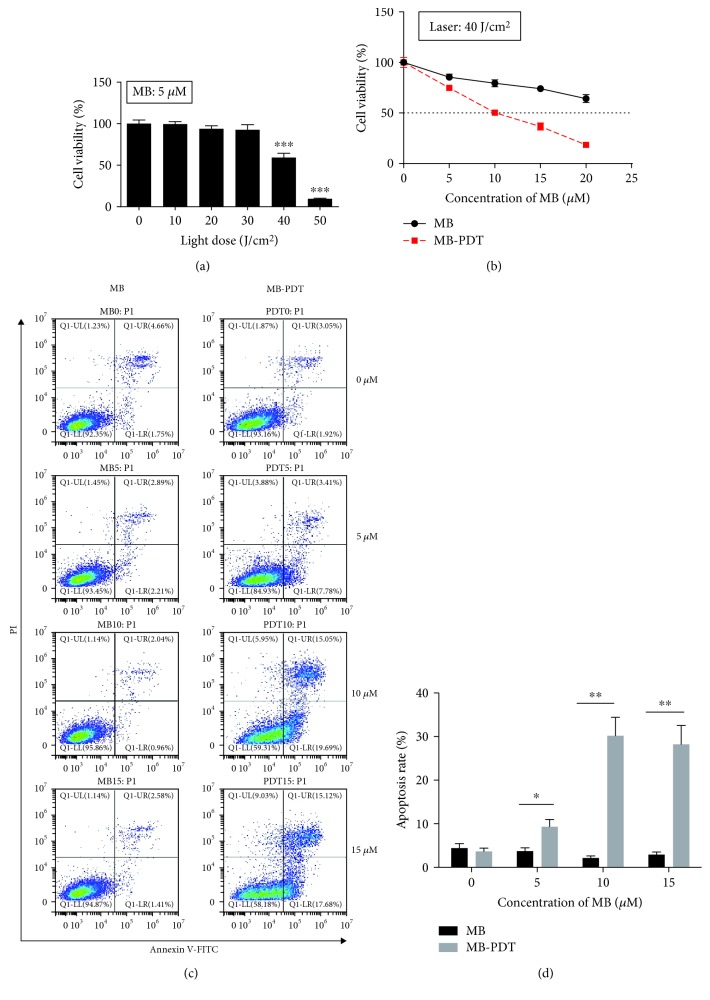
MB-PDT induced apoptosis in THP-1 macrophages. (a) The survival rates of THP-1 macrophages following different laser irradiation doses (ranging from 0 to 50 J/cm^2^) with 5 *μ*M MB. (b) The survival rates of THP-1 macrophages following 40 J/cm^2^ laser irradiation with different MB concentrations (0–20 *μ*M). The results are expressed as the percentage of viable cells compared with medium alone-treated controls. (c) The apoptosis rate of THP-1 macrophages following photosensitization experiments (MB ranging from 0 to 15 *μ*M with or without 40 J/cm^2^ irradiation) was assessed at 6 h posttreatment by Annexin V-FITC/PI binding and measured by flow cytometry analysis. The numbers indicate the percentage of cells in each quadrant. Specific statistical analyses are presented in (d). The results are expressed as the percentage of apoptotic cells (mean ± SD, *n* = 3). ^∗^*P* < 0.05, ^∗∗^*P* < 0.01, and ^∗∗∗^*P* < 0.001.

The MTT assay demonstrated that cell viability decreased with increasing irradiation of the laser (Figure 1(a)), and cell viability was significantly decreased (*P* < 0.001) at a light dose of 40 J/cm^2^ (IC_50_ = 41.36 J/cm^2^) with 5 *μ*M MB. As shown in Figure 1(b), cell viability was not affected in the culture treated by the laser alone (MB 0 *μ*M, laser 40 J/cm^2^) compared with the control subgroup (MB 0 *μ*M, laser 0 J/cm^2^). The cytotoxic effect of MB-PDT was considerably greater than that of MB, and more than half of the cells (approximately 50.36%) were killed when treated by PDT with 10 *μ*M MB. As illustrated in Figures 1(c) and 1(d), MB-PDT induced THP-1 macrophage apoptosis more effectively than MB alone (34.74% vs. 3.00%). The apoptosis rate of the cells in the laser alone-treated group (MB 0 *μ*M, laser 40 J/cm^2^) was almost equal to that in the control subgroup (MB 0 *μ*M, laser 0 J/cm^2^). Additionally, as the MB concentration increased, the apoptosis rate of the cells in the MB group did not obviously change, whereas the rate in the MB-PDT group increased. No significant difference (*P* > 0.05) in the apoptosis rate was observed between doses of 10 and 15 *μ*M in the MB-PDT group, and 10 *μ*M MB achieved the peak effect. When the concentration of MB was greater than 10 *μ*M, MB-PDT did not cause a larger number of macrophages to undergo apoptosis. Therefore, the application of 10 *μ*M MB and 40 J/cm^2^ laser irradiation represented the optimal conditions under which PDT maximized cell apoptosis.

### 3.2. ROS Produced by MB-PDT Played an Essential Role in Initiating Apoptotic Cell Death

**Figure 2:**
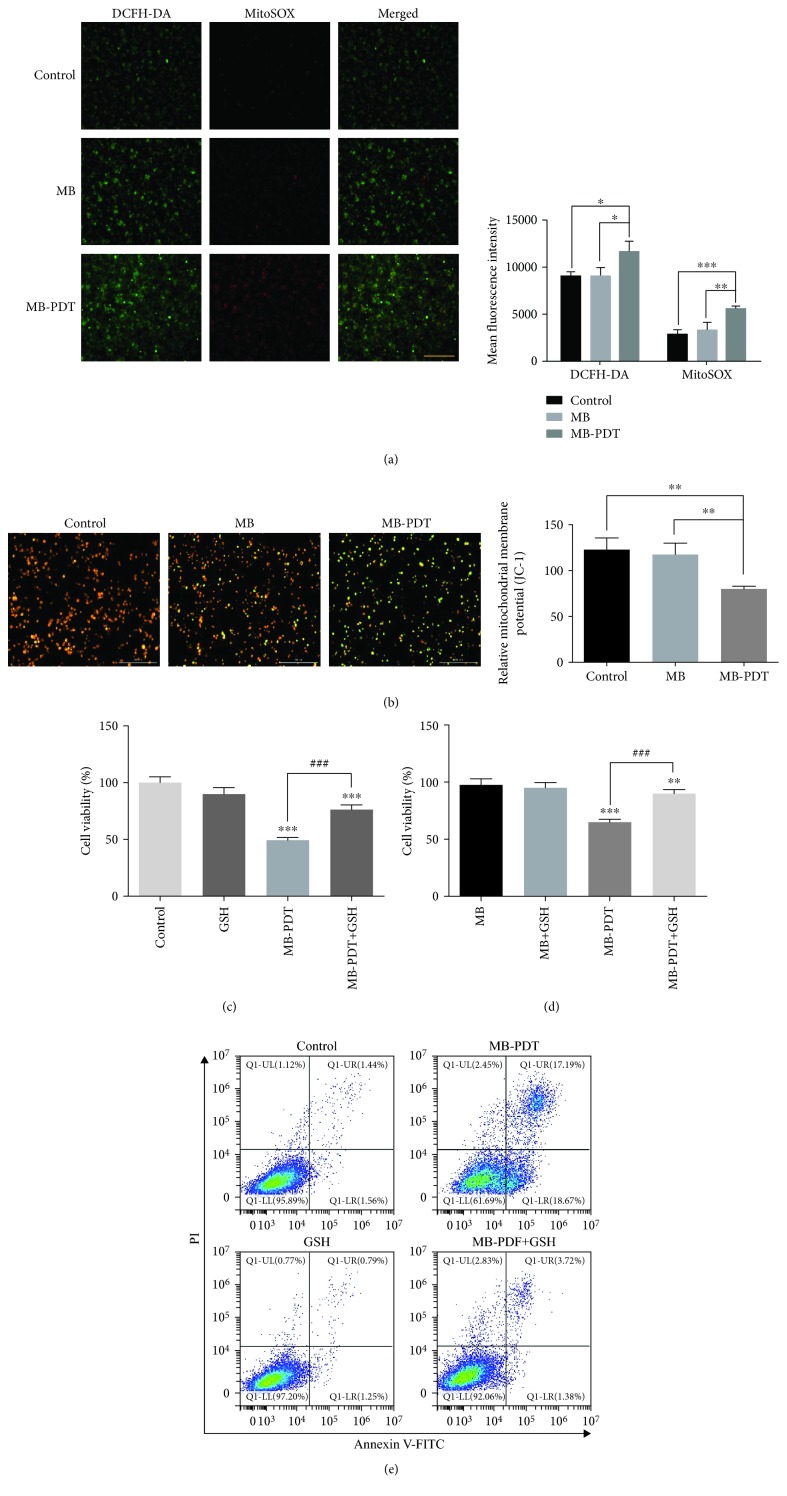
Mitochondrial ROS burst and MMP loss in THP-1 macrophages induced by MB-PDT. (a) Fluorescent photomicrographs of THP-1 macrophages stained with DCFH-DA and MitoSOX depicting increases in intracellular and mitochondrial ROS. The results are expressed as the mean fluorescence intensity of macrophages. (b) Fluorescent photomicrographs of THP-1 macrophages stained with JC-1 to depict the MMP. The results are expressed as the relative mitochondrial membrane potential. (c, d) Cell viability was assessed at 24 h in THP-1 macrophages treated with 20 mM GSH for 1 h, followed by treatment with MB-PDT. The results are expressed as the percentage of viable cells compared with medium alone-treated or MB alone-treated controls (mean ± SD, *n* = 3). (e) Apoptosis was assessed at 6 h in THP-1 macrophages treated with 20 mM GSH for 1 h, followed by treatment with MB-PDT. The results are expressed as the percentage of apoptotic cells. ^∗^*P* < 0.05, ^∗∗^*P* < 0.01, and ^∗∗∗^*P* < 0.001. Scale bar = 0.2 mm. Abbreviations: MB-PDT: methylene blue-mediated photodynamic therapy; DCFH-DA: 2′-7′-dichlorofluorescein diacetate.

As shown in Figure 2(a), intracellular and mitochondrial ROS levels in the MB-PDT group were higher than those in the control and MB groups. The increased fluorescence intensities of intracellular ROS and mitochondrial ROS detected by DCFH-DA/MitoSOX probes were 2,599.08 ± 928.68 au and 2,965.74 ± 50.84 au, respectively, in the MB-PDT group (vs. the control group). As shown in Figure 2(b), normal THP-1 macrophages exhibited red-orange fluorescence. MB alone did not trigger an MMP change, whereas THP-1 macrophages developed a diffuse green staining pattern indicative of decreased MMP after PDT. Moreover, we found that MB-PDT exhibited reduced cell-killing efficacy when we pretreated THP-1 macrophages with an antioxidant (exogenous GSH) (Figures 2(c) and 2(d)). Consistent with the above results, the apoptosis ratio changed from 35.86% to 5.10%, indicating that the addition of exogenous GSH almost completely prevented the apoptotic cell death induced by MB-PDT (Figure 2(e)).

### 3.3. MB-PDT Induced Apoptosis via the Intrinsic Mitochondrial Pathway

**Figure 3:**
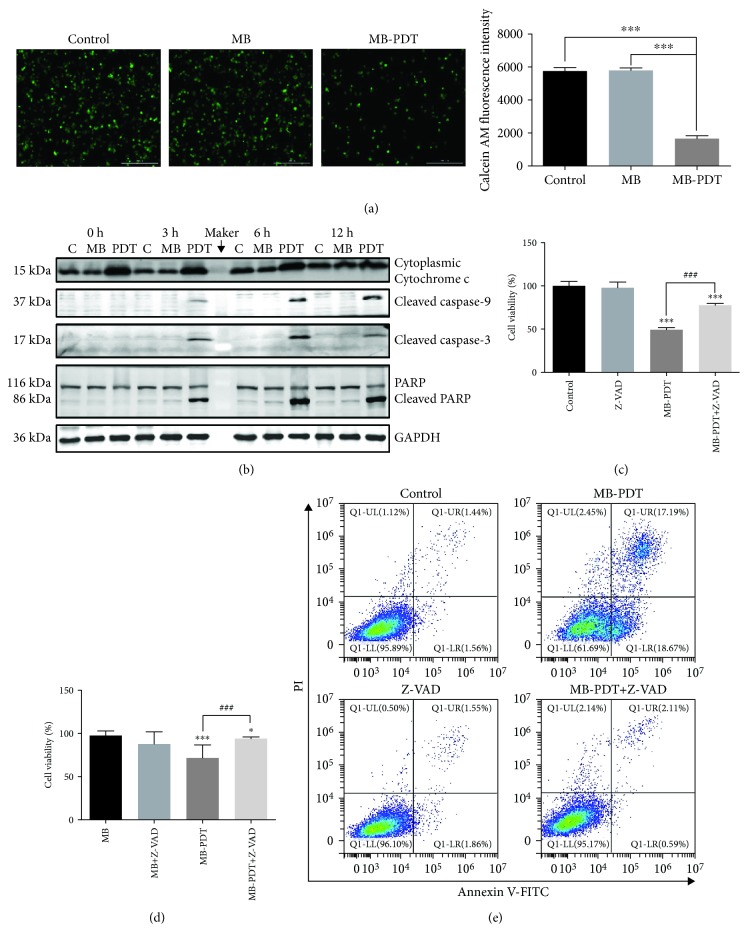
Release of cytochrome c via opening of the MPTP initiated apoptosis through the intrinsic apoptotic pathway. (a) Fluorescent photomicrograph of MPTP activation via calcein AM/CoCl_2_+ labeling. The results are expressed as the calcein AM fluorescence intensity. (b) Time-dependent apoptosis was measured in THP-1 macrophages. For this analysis, macrophages were treated with MB-PDT (10 *μ*M, 40 J/cm^2^) and then harvested at 0, 3, 6, and 12 h post-PDT. The blots were probed with specific antibodies as shown. (c, d) Cell viability was assessed at 24 h in THP-1 macrophages treated with 50 *μ*M Z-VAD-FMK for 1 h, followed by treatment with MB-PDT. The results are expressed as the percentage of viable cells compared with medium alone-treated or MB alone-treated controls (mean ± SD, *n* = 3). (e) Apoptosis was assessed at 6 h in THP-1 macrophages treated with 50 *μ*M Z-VAD-FMK for 1 h, followed by treatment with MB-PDT. The results are expressed as the percentage of apoptotic cells. ^∗^*P* < 0.05, ^∗∗^*P* < 0.01, and ^∗∗∗^*P* < 0.001. Scale bar = 0.2 mm.

To determine whether the MPTP participated in the apoptotic mechanism induced by MB-PDT, changes in MPTP activity were assessed by calcein AM staining. The results (Figure 3(a)) indicate that the green fluorescence intensity was lowest in the MB-PDT group, which revealed that MB-PDT induced MPTP opening. In addition, as shown in Figure 3(b), cytosolic cytochrome c expression levels were upregulated after MB-PDT treatment, which was released from mitochondria via MPTP opening. Consistently, Western blot assays showed a large increase in activated caspase-9. Moreover, cleaved caspase-3 and cleaved PARP were detected at 3 h in treated cells, and their expression levels were highest at 6 h, corroborating the induction of apoptosis of THP-1 macrophages by MB-PDT. To further confirm that MB-PDT-induced cell death was apoptotic and caspase-dependent, we treated cells with MB-PDT (10 *μ*M) in the presence or absence of Z-VAD-FMK (50 *μ*M), a pan-caspase inhibitor. Compared with the MB-PDT group, the addition of Z-VAD-FMK markedly reduced the cytotoxic and apoptotic response (Figures 3(c)–3(e)).

### 3.4. MB-PDT Induced Apoptosis of Macrophages in Rats with Periodontitis

**Figure 4:**
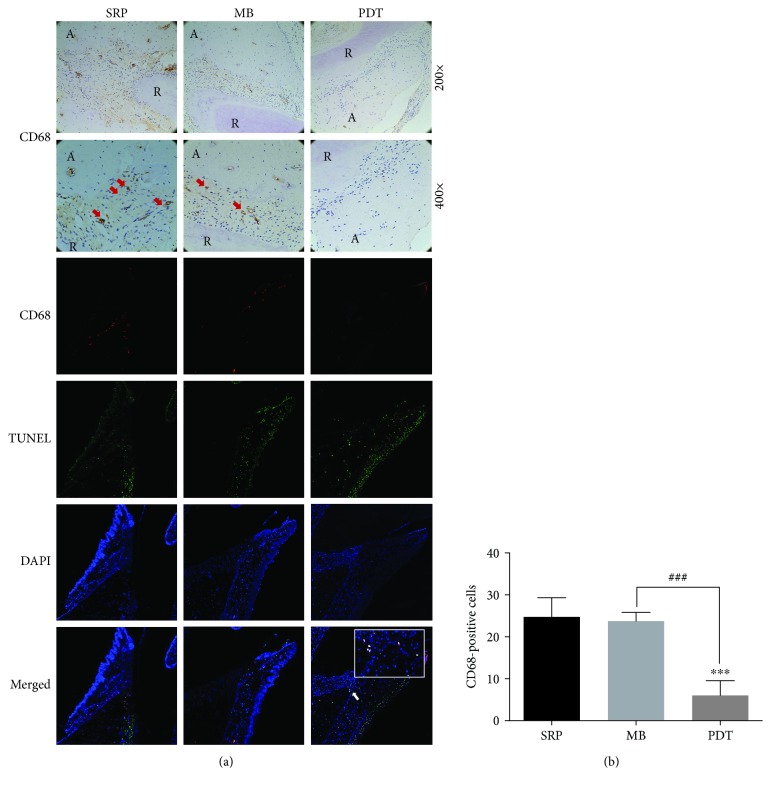
Immunohistochemical and TUNEL staining of periodontal CD68 macrophages in rats. (a) Representative images of coronal sections of maxillary first molars from SD rats inoculated intraorally with Pg ATCC 33277 and Fn ATCC 25586 and probed with anti-rat CD68 mAb. Macrophages (CD68^+^ cells) in the periodontium are indicated by red arrows (original magnification 200x/400x). Apoptotic macrophages (CD68^+^ TUNEL^+^ cells) in the periodontium are indicated by white arrows (original magnification 100x). (b) CD68-positive cells are expressed as the mean ± SD (*n* = 8). ^∗^*P* < 0.05, ^∗∗^*P* < 0.01, and ^∗∗∗^*P* < 0.001. Abbreviations: R: root; A: alveolar bone.

To further evaluate whether MB-PDT affected macrophages *in vivo*, immunohistochemistry and TUNEL staining were used to detect the amount of macrophage infiltration and whether apoptotic macrophages occurred in periodontal tissues of SD rats after MB-PDT. As shown in Figure 4, the number of infiltrative macrophages in the MB-PDT group was reduced compared with those in the SRP group and the MB group, whereas apoptotic macrophages were observed in the MB-PDT group but not in the SRP and MB groups.

### 3.5. MB-PDT Reduced Bone Resorption and Decreased Inflammatory Factor Levels

**Figure 5:**
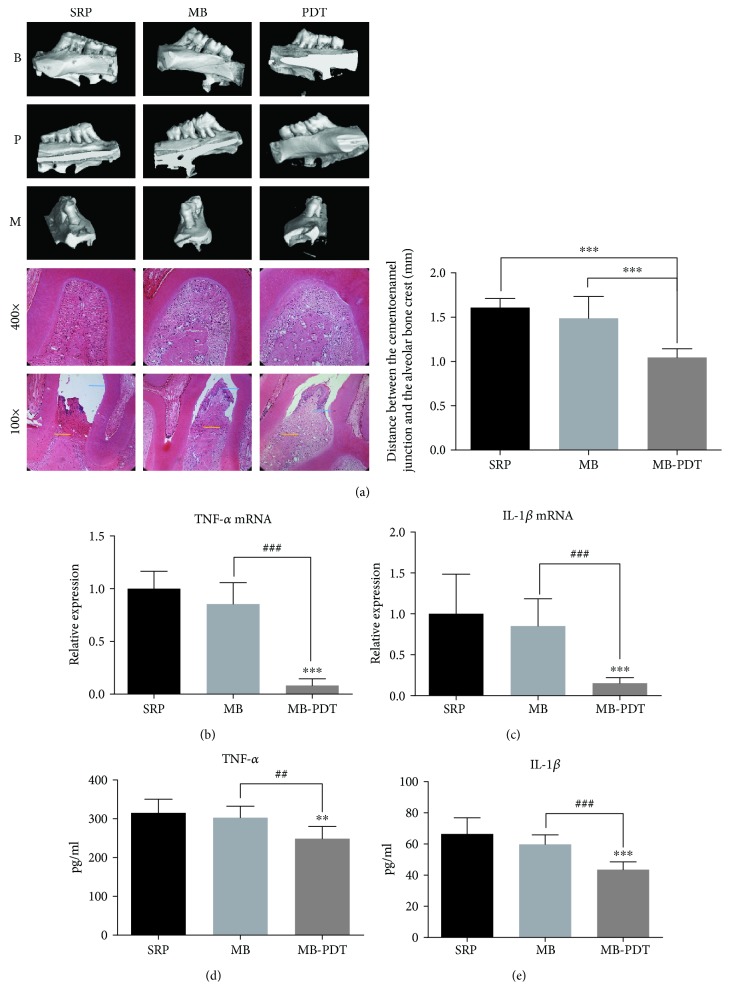
MB-PDT prevents ligature-induced alveolar bone resorption and reduces inflammation in SD rats with periodontitis. (a) Results of micro-CT and HE. HE revealed bone loss in the furcation region and distal root of the maxillary first molar. Scale bars of micro-CT images, 1 mm. The average distance from the cementoenamel junction to the alveolar bone crest on the mesial surface of the maxillary first molar was calculated. Blue lines on HE images: horizon of the cementoenamel junction; yellow lines: alveolar bone crest. (b, c) TNF-*α* and IL-1*β* mRNA expression levels in the periodontium were measured by qPCR. (d, e) The levels of TNF-*α* and IL-1*β* in the periodontium were measured by ELISA. All results are expressed as the mean ± SD (*n* = 8). ^∗^*P* < 0.05, ^∗∗^*P* < 0.01, and ^∗∗∗^*P* < 0.001. Abbreviations: B: buccal surface; P: palatal surface; M: mesial surface.

Bone loss was measured between the cementoenamel junction and the alveolar bone crest (Figure 5(a)). MB-PDT significantly reduced alveolar bone loss in rats with periodontitis compared to that in rats treated with SRP or MB (*P* < 0.001) (1.05 ± 0.03, 1.60 ± 0.02, and 1.49 ± 0.09 mm, respectively). Histological analysis showed that bone loss in the furcation region and distal root of the maxillary first molar in the MB-PDT group was lower than that in the SRP group and the MB group. We further analyzed periodontitis-related mRNA expression in periodontal tissues of experimental animals in all three groups in a short-term experiment (Figures 5(b) and 5(c)). TNF-*α* and IL-1*β* mRNA expression was significantly reduced in the MB-PDT group compared to the SRP and MB groups (*P* < 0.001). Moreover, ELISA also detected reduced inflammatory factors in the MB-PDT group compared to the SRP and MB groups (*P* < 0.01), but no significant difference was noted between the SRP and MB groups (Figures 5(d) and 5(e)).

## 4. Discussion

In this study, our hypothesis that MB-PDT can induce macrophage apoptosis both *in vivo* and *in vitro* was confirmed through experiments. We observed clear effects of SRP with MB-PDT on reducing inflammation in periodontal tissues and inhibiting bone absorption *in vivo*. The mechanism of macrophage apoptosis induced by MB-PDT was also demonstrated.

Recently, numerous patients with periodontitis have experienced benefits from undergoing photodynamic therapy [31]. The free oxygen radicals generated by PDT are lethal against pathogenic microbes, such as *P. gingivalis*, *Tannerella forsythia*, and *Treponema denticola* [32, 33]. Seguier et al. [34] loaded metal phthalocyanines with two different drug delivery systems (DDS; liposomes and nanoemulsions) and found that liposome photodynamic therapy reduced the number of macrophages in the gingiva of patients with chronic periodontitis. In another study, Lam et al. [35] used clodronate liposomes to deplete macrophages, which resulted in a significant reduction in macrophage infiltration of gingival, submandibular lymph node tissues, and *P. gingivalis*-induced bone resorption compared with controls in BALB/c and C57BL/6 mice. These studies are consistent with our research. In our study, all rats with periodontitis first underwent SRP and were then treated by MB-PDT or MB. SRP was the most important method of basic periodontal therapy, which could remove most of the plaque and calculi from the crown and root surfaces of teeth [36]. We found that when the periodontium of SD rats was treated with MB-PDT after SRP, less macrophage infiltration and more macrophage apoptosis than those in rats treated by SRP alone were noted. Additionally, we observed that less bone loss occurred and that there were lower levels of inflammatory factors (IL-1*β* and TNF-*α*) in MB-PDT rats. Therefore, we suggested that MB-PDT could not only alleviate periodontitis through its antimicrobial effect [37] but also inhibit the progression of periodontitis by inducing apoptosis of overinfiltrated macrophages. However, we could not quantitatively differentiate whether MB-PDT impeded periodontitis via macrophage apoptosis or bacterial inactivation since SRP could not eliminate all periodontal pathogens in the present study.

PDT must be based on three components to be effective: photosensitizers, light, and oxygen, none of which can be excluded. Our experimental results consistently show that laser application alone does not impair cell viability and MB application alone does not damage cells when the number of cells is high (10^6^/well). Meanwhile, we demonstrated that MB alone showed no significant treatment effect compared with SRP treatment alone *in vivo*. The commercial phenothiazine dye MB is an effective photosensitizing agent for the inactivation of pathogenic organisms, including viruses, bacteria, and yeast [38]. With laser activation, MB can produce a variety of ROS through type I and type II reactions, including singlet oxygen molecules (^1^O_2_), superoxide anion radicals (O_2_^·-^), and hydroxyl radicals (OH^·^) [39]. In addition, one study illustrated that hydroxyl radicals and superoxide decreased cell viability, whereas ^1^O_2_ acted as a trigger for imbalanced ROS generation in THP-1 macrophages after PDT [28]. Since singlet oxygen generation in THP-1 macrophages due to MB-PDT could not be detected after irradiation had ended, verifying whether MB-PDT could change the levels of intracellular and mitochondrial ROS detected by DCFH-DA (DCFH-DA does not detect ^1^O_2_ but reacts with hydroxyl radicals) and MitoSOX (MitoSOX detects superoxide in mitochondria) was a reasonable task. This experimental protocol was consistent with published papers [26, 28, 40]. Our study demonstrated that after MB-PDT, both intracellular and mitochondrial ROS were generated. MB produced almost equal levels of ROS in mitochondria compared to intracellular ROS in THP-1 macrophages after excitation combined with light, and the mitochondrial ROS level in the MB-PDT group was approximately twice as high as that in the control group (Figure 2(a)). Some previous studies showed that when incubated with a suspension of mitochondria, MB actively bound to this organelle and entered the matrix [39]. Mitochondrial cytolocalization of MB derivatives had also been confirmed by another study [41]. These data indicate that MB has an affinity for mitochondria.

**Figure 6:**
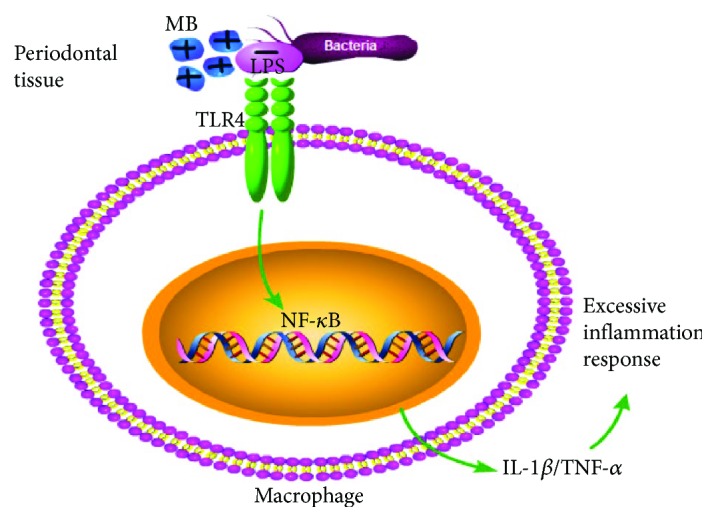
MB-PDT targeted macrophages through electrostatic attraction. MB was cationic and could be attracted by negatively charged bacterial LPS, which made contact with TLR4 in macrophages when periodontitis developed. Therefore, MB could aggregate in macrophages.

Pillusky [42] found that MB-PDT as an adjunct to SRP could induce a systemic protective response against periodontitis-induced oxidative stress and recover gingival collagen, indicating that MB-PDT is noninvasive and exhibits specificity for target cells [43]. MB could bind to the polyphosphates of the outside membrane and cause molecular damage to lipids and proteins, including membrane-bound enzymes [44]. Additionally, macrophages can be activated by a bacterial lipopolysaccharide (LPS), such as Pg LPS, a “classic” TLR4 ligand [45]. Therefore, we hypothesized that MB could target macrophages by binding to LPS, which makes contact with TLR4 in macrophages when periodontitis develops (Figure 6).

**Figure 7:**
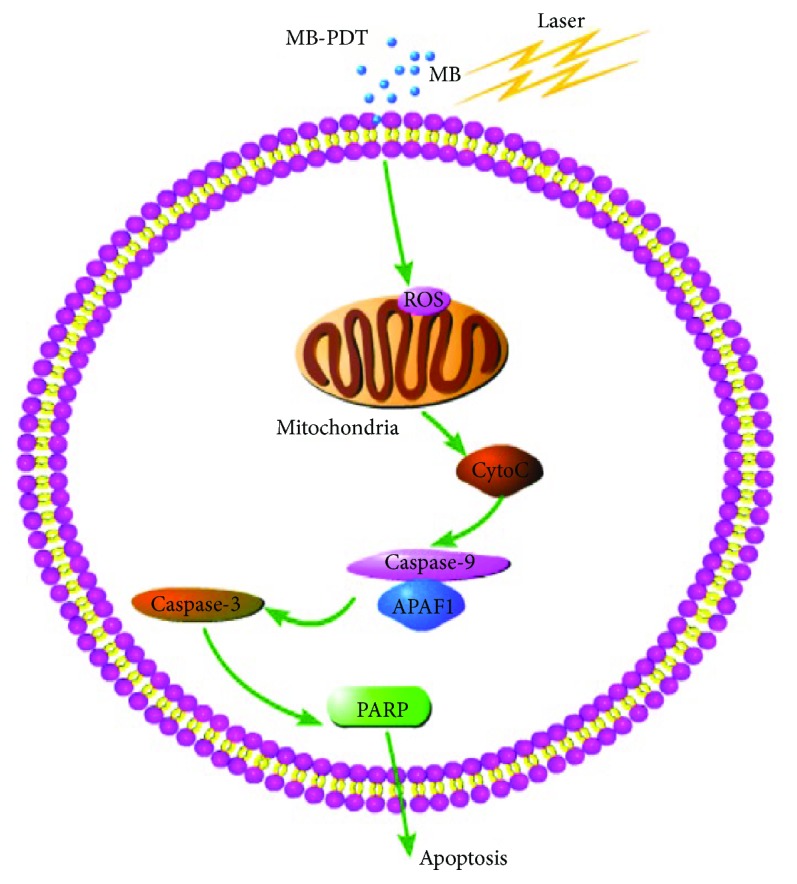
MB-PDT mediated the induction of apoptosis by the mitochondria-dependent pathway. MB seemed to selectively bind to mitochondria, where it produced ROS after excitation in combination with light. Then, ROS acted on mitochondria to induce an imbalance between ROS production and antioxidant capacity. Consequently, MB-PDT-mediated oxidative stress caused impairment of mitochondrial integrity and function, resulting in cytochrome c release and loss of mitochondrial membrane potential, which ultimately led to apoptotic cell death.

After PDT, three types of cell death were observed: apoptosis, necrosis, and autophagy. Apoptosis is an active gene-mediated process that automatically terminates life and is also the most common form of cell death caused by PDT. One study on the efficacy of MB-PDT in controlling oral lichen planus (OLP) suggested that PDT may exert immunomodulatory effects and induce apoptosis in hyperproliferating inflammatory cells, which are present in psoriasis and lichen planus [46]. Our results also suggested that MB-PDT killed THP-1 macrophages through an apoptotic process both *in vitro* and *in vivo*. There are three main pathways involved in PDT-induced apoptosis: (1) the death receptor-mediated apoptotic pathway, namely, the exogenous pathway; (2) the mitochondria-mediated intrinsic apoptotic pathway; and (3) induced release of Ca^2+^ from the endoplasmic reticulum via direct activation of phospholipase C (PLC) on the plasmalemma [47–49]. In the first pathway, caspase-8 is activated after the death receptor is stimulated by external stimuli to induce apoptosis. Mitochondrial ROS generation and MMP loss are known to cooperatively lead to mitochondrial membrane integrity disruption, consequently allowing the release of proapoptotic proteins [50]. In the second pathway, mitochondrial function is impaired, causing cytochrome c to be released into the cytoplasm, thus activating caspase-9 and inducing apoptosis. In these two pathways, both caspase-8 and caspase-9 activate the caspase effector factor caspase-3. Then, caspase-3 induces apoptosis. Consistent with our presumption, our results showed that MB-PDT treatment activated caspase-9, caspase-3, and PARP proteins. Conversely, addition of thexogenous antioxidant glutathione and the pan-caspase inhibitor Z-VAD-FMK markedly reduced the apoptotic response in macrophages. These findings revealed that MB-PDT induced apoptosis in THP-1 macrophages via the mitochondrial caspase pathway, which was activated by the ROS following MB-PDT (Figure 7). One study indicated that persistent depolarization was required for proapoptotic mitochondrial network abnormalities, although depolarization itself did not cause mitochondrial fragmentation and clustering [51]. As the polarization of mitochondria plays an essential role in the progression from nonapoptotic to proapoptotic mitochondrial network abnormalities, further studies are needed to discover which depolarization elicits these effects.

One limitation of the present study was that we did not specifically study which type of polarized macrophages, M1 or M2, was apoptotic after MB-PDT. The terms M1 and M2, which were proposed in 2000 by Mills et al., have long been used to define the “supposed” major subsets of macrophages [52]. Basically, M1 and M2 responses exemplify the opposing activities of proinflammation and anti-inflammation [53]. However, a study showed that periodontitis induced an M1-like specific signature with high levels of TNF-*α* and IL-6 through gene expression profiling in circulating monocytes in ligature-induced experimental periodontitis models, indicating that an M1-like phenotype of macrophages is induced by periodontitis [54]. Thus, based on our experimental results *in vivo*, MB-PDT may alleviate periodontitis by inducing apoptosis of M1 macrophages, which requires further verification in the absence of its antibacterial effects. Moreover, we should also detect whether MB-PDT can induce transformation of M1 macrophages to M2 macrophages given that M2 macrophages secrete anti-inflammatory factors [55].

Above all, we intend to use upconversion nanoparticles coated with an M1 macrophage-specific antibody to increase the targeting ability of photosensitizers to M1 macrophages infiltrated in inflammatory periodontal tissues in the near future.

## 5. Conclusion

MB-PDT could induce macrophage apoptosis *in vitro* and in rats with periodontitis. This may be another way for MB-PDT to relieve periodontitis in addition to its antimicrobial effect. Meanwhile, MB-PDT induced apoptosis in THP-1 macrophages via the mitochondrial caspase pathway.

## Figures and Tables

**Table 1:** Primers used for real-time PCR.

Genes	Forward primer 5′-3′	Reverse primer 5′-3′
GAPDH	ACA GCA ACA GGG TGG TGG AC	TTT GAG GGT GCA GCG AAC TT
TNF-*α*	GACCCTCACACTCAGATCATCTTCT	TGCTACGACGTGGGCTACG
IL-1*β*	GCACAGTTCCCCAACTGGTA	AAGACACGGGTTCCATGGTG

## Data Availability

The data used to support the findings of this study are available from the corresponding authors upon request.
